# Supplementary material for: Acute warming tolerance (CTmax) in zebrafish (Danio rerio) appears unaffected by changes in water salinity
Source: PeerJ. 2024 Jun 26;12:e17343. doi: 10.7717/peerj.17343 (PMC11214424; doi:10.7717/peerj.17343)
Supplement: Supplemental Information 1 — Overview of studies reporting tests of CTmax under different salinity regimes. Shows for each study the CTmax warming rate (Rate), the range of acclimation temperatures used (Acclimation range), the range of salinities used (Salinity range), the salinity ranges where these species are observed in the wild (Natural. salinity range), how long the fish got time to acclimate to their water salinities before testing CTmax (Salinity acclimation time), and what effect increasing salinity had on CTmax (Effect on CTmax). Some natural salinity ranges are gathered from independent studies (sources noted below table). [file peerj-12-17343-s001.docx]

**Supplementary table 1
Salinity and CT_max_ literature review.**

Overview of studies reporting tests of CT_max_ under different salinity regimes. Shows for each study the CT_max_ warming rate (Rate), the range of acclimation temperatures used (Acclimation range), the range of salinities used (Salinity range), the salinity ranges where these species are observed in the wild (Natural. salinity range), how long the fish got time to acclimate to their water salinities before testing CT_max_ (Salinity acclimation time), and what effect increasing salinity had on CT_max_ (Effect on CT_max_). Some natural salinity ranges are gathered from independent studies (sources noted below table).

| **Study/Species** | **Ramping Rate** | **Temperature range** | **Salinity range** | **Natural salinity range** | **Salinity acclimation time** | **Effect on CT_max_** |
| --- | --- | --- | --- | --- | --- | --- |
|  |  |  |  |  |  |  |
| **Shaughnessy & McCormick (2018)** |  |  |  |  |  |  |
| Brook trout (*Salvelinus fontinalis*) | **4 - 2°C/h** | **18°C** | **FW - 25 ppt** | **0-32 ppt^(1)^** | **0, 2, 5, 16d** | **Reduced** |
| Decreased CT_max_ during acclimation after acute transfer to seawater but restored to normal after 16 days. CT_max_ ramping rate 4°C/h first hour, then 2°C/h. FW = freshwater (salinity not specified). | | | | | | |
| **Morgan et al (2019)** |  |  |  |  |  |  |
| Zebrafish (*Danio rerio*) | **0.3°C/min** | **25-35°C** | **0.03-0.10 ppt** | **0.1-0.6 ppt^(2)^** | **Native** | **Reduced** |
| CT_max_ was negatively correlated with the water salinity of streams in wild zebrafish in India. | | | | | | |
| **Åsheim et al (this paper)** |  |  |  |  |  |  |
| Zebrafish (*Danio rerio*) | **0.3°C/min** | **26°C** | **0-5 ppt** | **0.1-0.6 ppt^(2)^** | **0, 10d** | **No effect** |
|  |  |  |  |  |  |  |
| **Hines et al (2019)** |  |  |  |  |  |  |
| Atlantic salmon (*Salmo salar*) | **0.1°C/min** | **12°C** | **2.5-30 ppt** | **0-14 ppt^(3)^** | **60, 400d** | **No effect** |
| Coho salmon (*Oncorhynchus kisutch*) | **0.1°C/min** | **12°C** | **2.5-30 ppt** | **0-28 ppt^(4)^** | **60, 400d** | **No effect** |
| Tested 60 and 400 days post-smoltification. | | | | | | |
| **Davis et al (2019)** |  |  |  |  |  |  |
| Delta smelt (*Hypomesus transpacificus*) | **0.3°C/min** | **16-20°C** | **2.4-12 ppt** | **0-18 ppt** | **0, 2, 4, 7d** | **No effect** |
| Mississippi Silverside (*Menidia beryllina*) | **0.3°C/min** | **16-20°C** | **2.4-12 ppt** | **0-35 ppt** | **0, 2, 4, 7d** | **No effect** |
| Largemouth bass (*Micropterus salmoides*) | **0.3°C/min** | **16-20°C** | **2.4-8 ppt** | **0-4 ppt** | **0, 2, 4, 7d** | **No effect** |
|  | | | | | | |
| **Haney & Walsh (2003)** |  |  |  |  |  |  |
| Blackbanded limia (*Limia melanonotata*) | **0.3°C/min** | **25-35°C** | **0-60 ppt** | **0-70 ppt** | **7-14d** | **Peak 30 ppt** |
|  | | | | | | |
| **Sardella, Sanmarti & Krütz (2008)** |  |  |  |  |  |  |
| Green sturgeon (*Acipenser medirostris*) | **0.3°C/min** | **18°C** | **1-24 ppt** | **8.8-32.1 ppt^(5)^** | **7d** | **Peak 10 ppt** |
|  | | | | | | |
| **King & Sardella (2017)** |  |  |  |  |  |  |
| Mozambique tilapia (*Oreochromis mossambicus*) | **0.3°C/min** | **20-37 °C** | **3-24 ppt** | **0-120 ppt^(6)^** | **28d** | **Increased*** |
| *24g/l acclimated fish had a larger thermal tolerance window. | | | | | | |
| **Metzger, Healy, & Schulte (2016)** |  |  |  |  |  |  |
| Three-spined stickleback (*Gasterosteus aculeatus*) | **0.3°C/min** | **18°C** | **2-20 ppt** | **0-30 ppt^(7)^** | **0, 14d** | **Increased** |
| Two populations (freshwater, seawater), tested at both salinities. Fish acclimated to higher salinity had higher CT_max_. Salinity acclimation affected expression of heat shock proteins during heat shock test. | | | | | | |

References for natural salinity ranges are 1: (McCormick et al., 1998), 2: (Spence et al., 2006), 3: (Otto & McInerney, 1970), 4: (Halfyard et al., 2012), 5: (Israel & Klimley, 2008), 6: (Whitfield & Blaber, 1979), 7: (DeFaveri et al., 2013)
