# Supplementary material for: Acute warming tolerance (CTmax) in zebrafish (Danio rerio) appears unaffected by changes in water salinity
Source: PeerJ. 2024 Jun 26;12:e17343. doi: 10.7717/peerj.17343 (PMC11214424; doi:10.7717/peerj.17343)
Supplement: Supplemental Information 2 — Overview of the time duration (minutes) from the start of salinity treatments until the test of CTmax for the 2-hour test group, for each group of fish tested for CTmax together. This is the Timeshort variable used in our model of CTmax (see Table 1 and statistical methods). [file peerj-12-17343-s002.docx]

**Supplementary table 2**

**Time in treatment.**

Overview of the time duration (minutes) from the start of salinity treatments until the test of CT_max_ for the 2-hour test group, for each group of fish tested for CT_max_ together. This is the Time_short_ variable used in our model of CT_max_ (see table 1 and statistical methods)

| **Tank** | **Salinity** | **Test box** | **Time (min)** |
| --- | --- | --- | --- |
| 4 | 0 | 1 | 95 |
| 4 | 0 | 2 | 101 |
| 7 | 0 | 1 | 153 |
| 7 | 0 | 2 | 159 |
| 2 | 0,5 | 1 | 128 |
| 2 | 0,5 | 2 | 128 |
| 5 | 0,5 | 1 | 118 |
| 5 | 0,5 | 2 | 154 |
| 1 | 1 | 1 | 143 |
| 1 | 1 | 2 | 143 |
| 6 | 1 | 1 | 149 |
| 6 | 1 | 2 | 154 |
| 3 | 5 | 1 | 153 |
| 3 | 5 | 2 | 157 |
| 8 | 5 | 1 | 156 |
| 8 | 5 | 2 | 164 |
